# Primary Tick-Borne Protozoan and Rickettsial Infections of Animals in Turkey

**DOI:** 10.3390/pathogens10020231

**Published:** 2021-02-19

**Authors:** Onur Ceylan, Xuenan Xuan, Ferda Sevinc

**Affiliations:** 1 Department of Parasitology, Faculty of Veterinary Medicine, Selcuk University, 42250 Konya, Turkey; onurceylan@selcuk.edu.tr; 2 National Research Center for Protozoan Diseases, Obihiro University of Agriculture and Veterinary Medicine, Obihiro, Hokkaido 080-8555, Japan; gen@obihiro.ac.jp

**Keywords:** domestic animals, livestock, tick-borne parasitic diseases, Turkey

## Abstract

Parasitic diseases caused by ticks constitute a barrier on global animal production, mainly in tropical and subtropical regions. As a country with a temperate and subtropical climate, Turkey has topography, climate, and pasture resources, and these resources are suitable for animal breeding and parasite–host–vector relationships throughout the country. This geography restricts the regulations on animal movements in the southeastern and eastern Anatolia because of the close contact with the neighboring states. The livestock resources in Turkey are regulated by strong foundations. Almost 30% of the agriculture-based gross domestic product is provided by the livestock industry. Parasitic diseases arising from ticks are endemic in Turkey, and they have a significant impact on the economy and animal health, particularly for ruminants. The main and economically-important tick-borne diseases (TBDs) suffered by animals include theileriosis, babesiosis, hepatozoonosis, and cytauxzoonosis caused by protozoa, and anaplasmosis and ehrlichiosis caused by rickettsiae. The most common hemoprotozoan and rickettsial agents are *Anaplasma marginale, Anaplasma ovis, Anaplasma phagocytophilum, Anaplasma platys, Babesia bigemina, Babesia caballi*, *Babesia ovis, Cytauxzoon felis, Ehrlichia canis, Hepatozoon canis, Theileria annulata* and *Theileria equi*. These diseases are basically controlled through treatment and measures for tick control. Vaccination can be performed for only tropical theileriosis caused in Turkey. We reviewed the studies published in domestic and international journals to gather epidemiological data regarding the major TBDs suffered by animals in Turkey.

## 1. Introduction

Located between 26°–45° eastern longitudes and 36°–42° northern latitudes, Turkey has a critically strategic location in the Mediterranean region, functioning as a bridge between the Europe and Asia. Turkey’s climate, topography, and forage resources make the country very suitable for animal agriculture. Additionally, Turkey’s temperate and subtropical climate characteristics ensure appropriate conditions for maintaining parasite–host–vector relationships. The regulation of animal movement is limited in the southeastern and eastern parts of Anatolia because of close geographical contact with neighboring countries. Generally, management systems are extensively and traditionally implemented for ruminants which typically graze in pastures during almost the whole year, usually from early days of spring to late periods of autumn. Conventional farms are mostly found in the eastern provinces while the modern farms have usually been constructed in the western provinces. Although the Turkish government grants subsidies to maintain the sector, traditional farming is on the decline because of high feed expenses and limitations regarding the access to pastures on common lands [1]. All factors associated with climate and geographic conditions and management systems leave the animals open to possible exposure to a variety of diseases [2,3].

The economy of Turkey is made of contemporary industry, commerce, and agriculture sectors. Thanks to the suitable climate, geographic conditions, rich soil sources and biological diversity, agriculture is one of the main sectors in the country. Though this sector tended to shrink following the 1980s, it is still a significant part of the current Turkish economy, accounting for 19.2% of the total employment in August 2020 [4]. Turkey has a remarkable foundation for livestock resources, and its share within the livestock sector of agricultural gross domestic product is approximately 30%. The main products of the Turkish livestock sector include poultry, beef, and veal. Poultry is the type of meat produced the most in the country. Most of the beef and veal is produced at traditional farms. Small ruminants represent the main livestock resource, with over 55 million in total animal numbers. Sheep constitute 57.98% of the total number of animals, while cattle represent 25.01%. Moreover, goats constitute 16.76%, and buffalo cover 0.25% of total livestock resource. Registered horses are solely present in state stud farms and jockey clubs. According to the report of the State Institute of Statistics, the number of horses was 131,497 in 2014 [1,4]. While the number of dogs and cats is unknown due to the absence of any compulsion regarding registration, these animals are believed to be plenty in Turkey.

Diseases arising from ticks cause significant medical and management issues in the farming sectors of Asian, African, and South American countries located in the tropical and subtropical regions of the globe. The geographic and climatic conditions and management systems in Turkey support the reproduction of ticks and emergence of TBDs. Relevant studies indicate that Turkey is an endemic location for TBDs, and various TBDs have been seen among domestic animals [2]. This study reviews epidemiological studies on frequent TBDs, including anaplasmosis, babesiosis, cytauxzoonosis, ehrlichiosis, hepatozoonosis, and theileriosis to emphasize the importance of these infections for animal health.

## 2. Ticks and Tick-Borne Diseases in Turkey

A total of 51 tick species, 43 from the Ixodidae family and 8 from the Argasidae family, have been identified in Turkey. The most important species are involved in nine genera including *Dermacentor*, *Haemaphysalis*, *Hyalomma*, *Ixodes*, *Rhipicephalus* (*Boophilus*), *Amblyomma, Argas*, *Otobius*, and *Ornithodoros*. The first five genera widely distributed throughout Turkey. The most prevalent species are *Rhipicephalus bursa, Rhipicephalus annulatus, Rhipicephalus sanguineus, Rhipicephalus turanicus, Hyalomma anatolicum, Hyalomma excavatum, Hyalomma marginatum, Dermacentor marginatus, Dermacentor niveus, Haemaphysalis parva, Haemaphysalis punctata*, and *Haemaphysalis sulcata*. The seasonal fluctuation of ticks depends on the genus, with *Rhipicephalus* and *Hyalomma* species found in spring, summer, and autumn, and *Dermacentor, Hemaphysalis, Ixodes*, and *Ornithodoros* species found in autumn, winter, and spring. The rate of tick-infested animals is usually > 20% of the herd [5,6,7,8,9,10,11,12,13].

Anaplasmosis, babesiosis, and theileriosis have been well-recognized TBDs of animals for many years, and remain as the most important diseases affecting livestock. Although the actual numbers of infections cannot be estimated from literature sources, the huge number of disease-specific drugs consumed during the tick activity seasons every year indicates the significance of these diseases on the economy and animal health.

## 3. Theileriosis

### 3.1. Bovine Theileriosis

Among several *Theileria* species, *Theileria annulata* and *Theileria parva* are the most pathogenic and financially critical species infecting cattle. The main TBD affecting cattle is tropical theileriosis resulting from *T. annulata* transmitted by *Hyalomma* in Turkey. Consequently, high morbidity and mortality occurs among the cattle in the entire country. Primary vectors of *T. annulata* are *Hyalomma anatolicum* and *H. detritum* but other *Hyalomma* species, including *H. marginatum, H. excavatum*, and *H. dromedarii*, can also transmit this hemoprotozoan agent. Benign *Theileria* species, *Theileria mutans* and *Theileria sergenti/buffeli/orientalis*, have been reported to be present in cattles from different locations of Turkey [14,15,16,17,18,19,20,21,22,23,24]. Discussions concerning the taxonomy of *T. sergenti/buffeli/orientalis* group has been continued for many years. These hemoparasites are classified as *T. orientalis* due to various biological characteristics [25,26]. As an emerging disease, oriental theileriosis, which is mainly characterized by hemolytic anemia, abortion, yield decrease, and pyrexia, can adversely affect cattle industry [27,28,29]. The molecular prevalence of *T. orientalis* has varied between 0.9% and 13.6% [14,18,23], and investigations conducted for determining the genotypes of *T. orientalis* revealed the presence of chitose (type 1) and buffeli (type 3) genotypes in Turkey [23,24].

**Figure 1:**
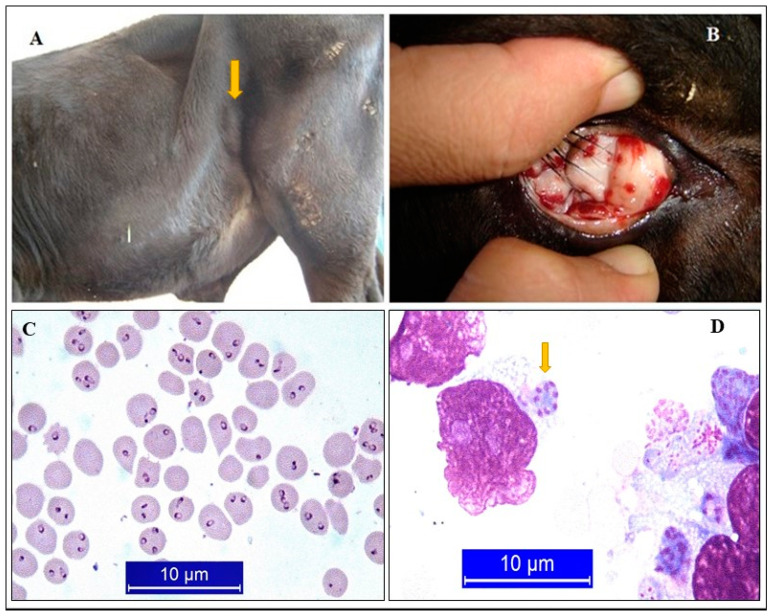
Certain clinical symptoms of tropical theileriosis. Swollen sub-iliac lymph node (**A**), Petechial hemorrhages in the conjunctiva (**B**), Giemsa-stained pyroplasmic forms of *T. annulata* (**C**), Schizont of *T. annulata* in a lymphoid cell (**D**) [30].

Tropical theileriosis is more significant for pure breeds compared to the domestic and cross-breeds. Cattle that are brought from other countries and unvaccinated are most susceptible to *T. annulata* infection. The mortality rate is >70% among them, while the same rate is < 45% in cross-bred indigenous cattle [10,19]. The disease usually occurs in a seasonal form, from May to September, and with peaks in July. Acute theileriosis is accompanied by anemia, fever, petechial hemorrhages on mucosal membranes, enlarged lymph nodes, labored respiration, abortion, decreased milk production, and death (Figure 1) [30].

A mixture of buparvaquone and oxytetracycline has been utilized to provide treatment to sick animals. Early diagnosis plays a key role in the effectiveness of the treatment. In cases with a parasitemia level > 20%, the treatment regime has also been supported with hematinic and anti-inflammatory agents. Blood transfusions have been necessary for severely anemic animals. A live-attenuated *T. annulata* schizont cell-culture-derived vaccine produced by the Ministry of Agriculture has been used to achieve disease control [8,10,20,31]; however, certain clinical reactions and deaths arising from the disease have been reported in vaccinated cattle [32,33].

Under field conditions, the significant factors affecting the severity of clinical infections are animal breed, management systems, and vaccination status. In an epidemiological study performed in the Cappadocia area that comprises a majority of the Central Anatolia Region, a total of 554 cattle, 62% of which were vaccinated against tropical theileriosis, were monitored for occurrences of clinical disease from April 1999 to November 2001, including three disease seasons. The findings indicated that the morbidity and mortality rate was higher among unvaccinated compared to vaccinated animals, in grazed compared to semi-grazed animals, and in pure breeds compared to cross or local breeds. The number of acute infections was 156 (27.61%), 86 of which died from tropical theileriosis. During the survey in the Cappadocia area, the total financial losses attributed to the infection were estimated to be 598,133 USD [19].

The diagnosis of theileriosis is mainly made through the microscopic examination of lymph and blood smears. In different parts of Turkey, the piroplasm forms of *T. annulata* in cattle have been detected upon the microscopic examination of Giemsa-stained blood smears at rates ranging from 2.3% to 60.5% [19,34,35]. Serological investigations performed by indirect fluorescent antibody test (IFAT) revealed the seroprevalence of *T. annulata* between 10% and 90%. Polymerase chain reaction (PCR) and reverse line blot assays showed that the molecular prevalence of *T. annulata* varied between 18.1% and 61.2% in cattle, and 12.6% and 46.9% in ticks [6,7,14,16,17,21,22,23,34,36,37,38]. Majority of the relevant studies are conducted in the Central and Eastern Anatolia Regions with high prevalence rates of tropical theileriosis. The results obtained from the Black Sea Region indicate the prevalence of hemoparasites to be lower than those of previously mentioned regions [17].

Epidemiological data regarding the vectors of bovine theileriosis were collected from surveys conducted under laboratory and field conditions. Inci et al. [35] found *T. annulata* infection to be at a rate of 17.49% in *H. anatolicum’* salivary glands by microscopy. Sayin et al. [39] examined the developmental stages of *T. annulata* in four *Hyalomma* spp. which fed on experimentally infected calves. They investigated the impacts of tick sex and species on the intensity of *T. annulata* infection in the tick salivary glands. The infection rates were almost equally high in all ticks, but the mean intensity of the infection was higher in female ticks. Aktas et al. [37] examined the intensity and prevalence of *Theileria* infections in *Hyalomma* ticks collected from shelters and cattle reared using the traditional management system in the eastern provinces. According to their results, 44% of cattle were infested with four *Hyalomma* species. *Hyalomma anatolicum* was the predominant tick with a rate of 63.1%, and the other species were *H. excavatum, H. detritum*, and *H. marginatum* with rates of 23.8%, 11.7%, and 0.6%, respectively. *Hyalomma anatolicum* displayed the highest infection rate. The ticks collected from shelters and cattles displayed the infection rates of 46.9% and 12.6%, respectively. Female ticks collected from cattle had a higher prevalence rate compared to male ticks, but the difference between the infection rates of female and male ticks collected from shelters was not significant. The mean intensity of infection was higher in female ticks collected from both shelters and cattle. In another research performed in the Central Anatolia region [7], 39% of cattle were infested with *Rhipicephalus annulatus, R. turanicus*, and *H. marginatum*, and 27.9% of 42 tick pools were found to be positive for hemoparasites including *Babesia bigemina, T. annulate*, and *Babesia* sp. The most common tick species was *R. annulatus*, and the most common hemoparasite was *B. bigemina*, followed by *T. annulata*.

### 3.2. Ovine Theileriosis

Many Theileria species (T. ovis, Theileria lestoquardi, T. luwenshuni, T. recondita, T. uilenbergi, T. separata, Theileria sp. OT1, and Theileria sp. OT3) are found in small ruminants. Among these, T. ovis, T. separata, and T. recondita are non-pathogenic species. The most pathogenic Theileria species are T. lestoquardi, T. luwenshuni, and T. uilenbergi. These species cause malignant theileriosis, while the others cause non-pathogenic theileriosis in goats and sheep [40,41,42,43,44]. Malignant ovine theileriosis causes death in sheep in the Middle East, South East Asia, the Mediterranean region, and the Indian subcontinent [45]. Theileria ovis is the most prevalent species for small ruminants in Turkey. While T. recondita and T. lestoquardi have been found, no associated clinical infections have been documented [31,46]. Among Theileria species, most of which are transmitted by Hyalomma spp. and Haemaphysalis spp. [47], T. luwenshuni, T. ovis, T. uilenbergi, [48,49] and some isolates such as Theileria sp. OT1, Theileria sp. OT3, and Theileria sp. MK [15,48,50,51,52] have been detected in goats and sheep in Turkey. Moreover, the DNA of T. annulata, a bovine Theileria species, has been described in goats and sheep in the country [52]. The results of epidemiological investigations indicated that the prevalence of T. ovis ranged from 9.2% to 67.96% in sheep, and 0% to 17.1% in goats in Turkey [15,49,50,52,53,54,55,56,57,58]. Rhipicephalus bursa plays a key role as a natural tick vector of T. ovis [53,59]. When compared to bovine theileriosis, small ruminant theileriosis has a minor impact on animal health in the country.

### 3.3. Cytauxzoonosis

As an emerging tick-borne hemoprotozoan infection of wild and domestic felids, cytauxzoonosis has been increasing in significance. The etiological agents of cytauxzoonosis are *Cytauxzoon* species which are apicomplexan parasites belonging to the order Piroplasmorida and the family Theileriidae. The disease in cats is characterized by anemia, dyspnea, inappetence, icterus, pyrexia, listlessness, and death [60,61,62]. *Cytauxzoon felis*, *Cytauxzoon manul*, and undetermined *Cytauxzoon* species which have minor genetic differences in 18S rRNA genes cause infections in domestic cats and wild felids [62]. These agents are transmitted to felid intermediate hosts by blood-feeding ticks harboring infective sporozoites of parasites [63,64]. Among these species, *C. felis* has a wide distribution and was detected in Turkey where the number of studies on feline hemoprotozoan and rickettsial diseases is restricted. *Cytauxzoon felis* infection in the cats of Van has been reported in only one study conducted in Van province of Turkey [65]. More detailed and comprehensive epidemiological surveys should be conducted to obtain data on feline cytauxzoonosis in Turkey.

## 4. Babesiosis

Babesiosis is a hemoprotozoan infection caused by *Babesia* species among humans and various domestic and wild animals. This disease is endemic, particularly in ruminants in tropical and subtropical zone countries. In regard to the number of clinical infections and deaths, ovine babesiosis has considerable economic and health significance for the Turkish livestock industry.

### 4.1. Bovine Babesiosis

**Figure 2:**
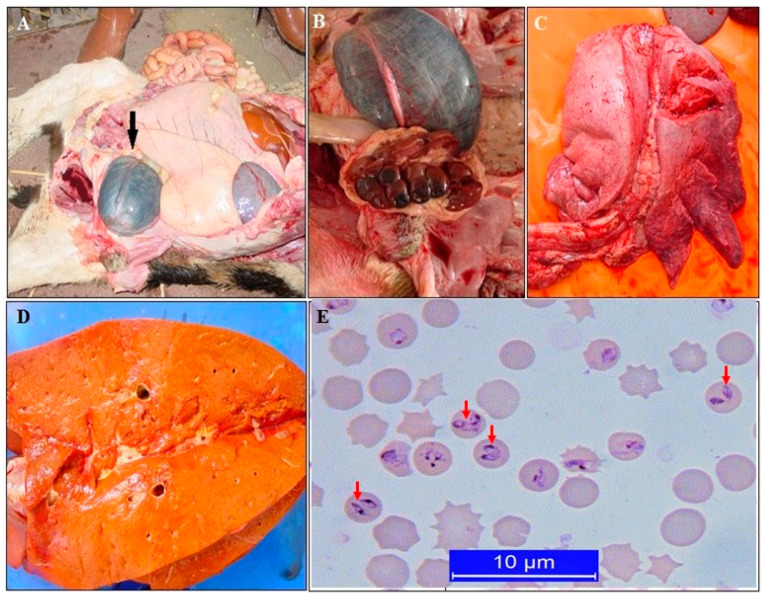
Post-mortem findings of bovine babesiosis caused by *Babesia bigemina*. Enlarged urinary bladder containing dark-colored urine (**A**), kidney degeneration (**B**), lung edema (**C**), jaundice of the liver (**D**), intra-erythrocytic merozoites on a Giemsa-stained blood smear (**E**) [30].

Bovine *Babesia* species are *B. bigemina, B. bovis, B. divergens, B. major* and *B. occultans*. These species are transmitted by the ticks belonging to the *Ixodes* and *Rhipicephalus* genera [3]. Most of the bovine babesioses cases occur owing to *B. bigemina* in Turkey [3,6,23,33,66,67] and are usually transmitted by *R. annulatus*. During an acute infection, the host becomes severely ill, generally suffering from severe anemia, high fever, hemoglobinuria, inappetence, and lethargy. In *B. bigemina* infections, parasitemia may reach a rate of 50% to 100%. Parasitemia of *B. bovis* is not as high as that of *B. bigemina*, but can be as high as 90% in impression smears prepared from brain tissue [68]. Death occurs following hypothermia accompanied by uremia with icterus in severe cases. The mortality rate is high in most acute babesiosis cases. Main post-mortem findings include an enlarged bladder with hemoglobinuric urine, icterus on mucosal membranes, and connective tissues, and jaundice of the liver (Figure 2) [30].

Imidocarb dipropionate has been administered as an anti-babesial drug to treat animals [69]. Live-attenuated vaccines have been used to control bovine babesiosis in many global locations; a single dose of vaccine ensures long-lasting protective immunity in cattle [70,71,72,73,74]. Nevertheless, there is no available vaccine against bovine babesiosis in Turkey [46,67] where *B. bigemina, B. bovis*, and *B. divergens* were detected through microscopy and serology while *B. major* was detected with PCR [75]. The prevalence of *B. bigemina* reportedly ranges from 0.6% to 53.07% depending on the geographic region [3,6,23,33,38,66,76]. Molecular studies have recently revealed the presence of *B. occultans* in Turkey. Aktas and Ozubek [77] determined the molecular prevalence of *B. occultans* as 3% in the Black Sea Region of the country. Moreover, the DNA of *B. occultans* were detected in *H. marginatum* and *R. turanicus* [78,79]. However, the role of these ticks in the effective transmission of *B. occultans* remains to be proved in Turkey [3]. Within the hemoparasites detected in ticks, *B. bigemina* has been reported as the predominant species in the central Turkey. The most prevalent tick species was *R. annulatus* at 26.37% followed by *H. marginatum* at 21.12% and *R. turanicus* at 18.7% [7]. While records on bovine babesiosis have been received regularly by veterinary practitioners throughout the disease seasons, the data is yet to be adequate to evaluate the number of cases and predict the economic impact on the livestock industry due to the absence of a case recording system.

### 4.2. Ovine Babesiosis

**Figure 3:**
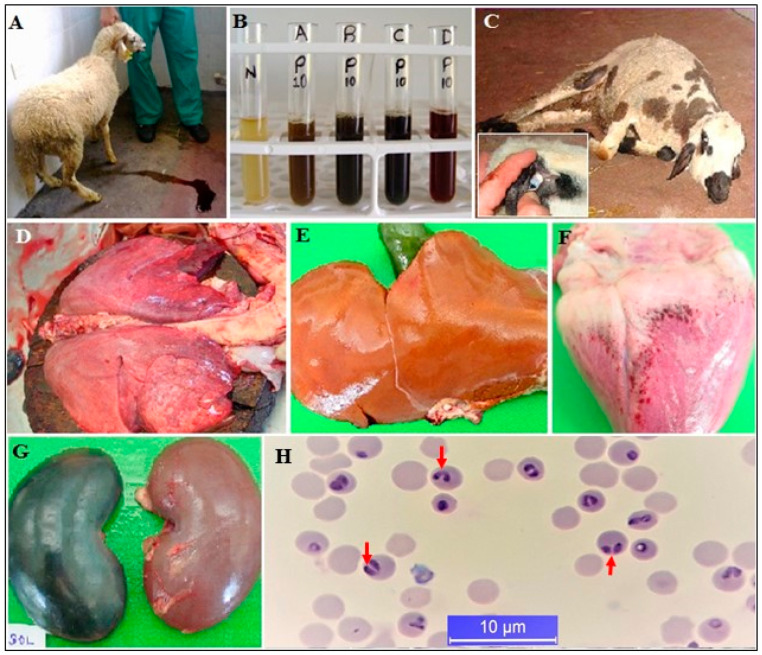
Post-mortem and clinical signs of ovine babesiosis. Hemoglobinuria, which is the most prominent clinical symptom of acute infection and anemia (**A**–**C**), lung edema (**D**), jaundice of the liver (**E**), petechial hemorrhages in the heart (**F**), kidney degeneration (**G**), and intra-erythrocytic merozoites on Giemsa-stained blood smears (**H**), ref. [30].

*Babesia* species causing ovine babesiosis are *Babesia ovis*, *Babesia crassa, Babesia foliata, Babesia motasi*, *Babesia taylori*, *Babesia* spp. Xinjiang, and *Babesia* spp. from China [80,81,82]. In a recent study, a new *Babesia* sp., which differs from the ovine *Babesia* species and genotypes registered in GenBank, was reported in goats in Turkey [83]. *Babesia ovis* is the most significant species infecting small ruminants in Africa, Asia, Europe, and the Far East. The geographic distribution of *Babesia* species shows parallelism that of their vectors, which are mostly *Haemaphysalis* and *Rhipicephalus* spp. in tropical and subtropical regions [42,81,84,85,86,87,88,89,90,91]. Babesiosis is the main TBD affecting small ruminants considered as the major livestock species of Turkey. Ovine babesiosis is an acute disease characterized by hemolytic anemia, fever, icterus, and hemoglobinuria in small ruminants (Figure 3) [30]. The disease emerges mostly as outbreaks in Turkey [92].

Sheep are the primary livestock resource, constituting 57.98% of the total livestock in Turkey [4]. Small ruminants are raised mostly under traditional systems. Grazing on pastures from spring to late autumn, the sheep are usually exposed to tick infestations. *Rhipicephalus* species is the common vectors of the disease in the country. As a seasonal disease, babesiosis is observed from April to October every year, most commonly in June and July [5,8,9,51,92]. *Babesia ovis* is highly pathogenic to sheep, and clinical cases are usually severe. In acute cases, pancytopenia characterized by anemia, leukopenia, and thrombocytopenia occurs in sick animals [81,84,85,86,87,92]. Untreated cases frequently result in death for some animals with severe infection despite treatment. Many recurrences may also take place after the treatment of animals with an anti-babesial drug. Even when acutely infected cases are treated with a specific drug, compensation for the abnormalities in the hematologic picture still takes a long time [92]. Serologic surveys indicate that serum samples collected from randomly-selected sheep have *B. ovis*-specific antibodies with a percentage ranging from 32% to 80% [8,75,93,94,95,96,97,98]. In a recent country-wide seroepidemiological study, the seroprevalence of *B. ovis* was determined as 49.16% by IFA test and 29.89% by rBoSA1-based enzyme-linked immunosorbent assay (ELISA). Ovine babesiosis has been observed to be particularly common in the Central, Southeastern, and Eastern Anatolia regions [99]. The molecular prevalence of *B. ovis* was determined to be between 0% and 21.42% in randomly selected sheep in Turkey [48,54,100,101,102,103]. However, Sevinc et al. [49] detected 70.81% molecular positivity in sheep showing clinical symptoms. Benedicto et al. [58] also detected high molecular positivity in tick-infested sheep (64.8%) and goats (53.6%) from Turkey. Making predictions regarding the current actual intensity of clinical cases is challenging considering the lack of a case recording system. Nevertheless, the results of a follow-up study performed in Konya located in the central part of Turkey reflect the importance of ovine babesiosis. A total of 122 acute infections (14.35%) were detected in only two sheep flocks between June and July in 2011, and 15 sheep died despite intensive treatment [92].

The control of ovine babesiosis is solely provided by chemotherapy and limited tick-control precautions. Imidocarb dipropionate has prophylactic efficiency as well as a therapeutic effect on the disease [69], therefore significant amounts of the agent are used every year. An immunoprophylactic control method against ovine babesiosis has not been developed. According to the relevant authorities, despite certain disadvantages, the attenuated parasites should be used to immunize the animals against TBDs as long as non-living parasite vaccines are absent [70,104,105]. The rapid passage of the virulent strain via susceptible splenectomized calves is one of the most extensively used methods for reducing the virulence of *B. bovis*. The literature displays limited attention to the immunoprophylaxis of *B. ovis* infection and no information regarding how many passages are required to attenuate a virulent *B. ovis* strain is available. Yeruham et al. [106] were unable to conduct more than three successive passages of *B. ovis* in splenectomized lambs since the third passage lamb had a very low level of parasitemia with no reduction in virulence. In a preliminary study [107], rapid blood passages were performed to reduce the virulence of *B. ovis* in 13 susceptible splenectomized lambs. The obtained results indicated the virulence of *B. ovis* was not eliminated after 12 successive passages. Therefore, alternative methods or further passages may be required to attenuate *B. ovis*.

Acute babesiosis is diagnosed depending on clinical signs of disease and the demonstration of the Giemsa-stained parasites in the erythrocytes by microscopy. Microscopical and clinical examinations are not sufficient for the diagnosis of subclinical infections. Immunological and molecular techniques have been employed to determine the subclinically infected animals [72,108,109]. A synthetically derived bovine *B. bovis* antigen has been utilized in an ELISA to detect anti-*B. ovis* antibodies for the immunodiagnosis of *B. ovis* [93,94,96,97]. IFAT is also being utilized in a small number of laboratories [69,98,99,110]. The IFA test has some disadvantages in that it is subjective, arduous, time-consuming, and requires well-experienced personnel. As diagnostic antigens, the immunoreactive proteins of certain *Babesia* species have widely been used in the immunodiagnosis of bovine, canine, and equine babesiosis [111,112,113,114]. In a recent study, the presence of five immunoreactive proteins of *B. ovis* was documented; however, further studies are still needed in terms of the purification of these proteins and their possible usage as diagnostic antigens [115]. Recombinant immunoreactive proteins have been utilized as antigen sources in quantitative diagnostic techniques and as vaccine candidates for the control of diseases. The results of a new research indicate that secreted antigen 1 of *B. ovis* (rBoSA1) is a promising diagnostic antigen that can be used for the development of serological assays for the diagnosis of ovine babesiosis [116]. Apart from rBoSA1, various immunoreactive proteins of *B. ovis* such as rBoSA2, BoSPD, and ovipain-2 have been produced [116,117,118,119]. These proteins are the strongest candidates to be used in future diagnostic kits and vaccine development. Recombinant BoSA1-based indirect ELISA was successfully used in a seroepidemiological study to determine the endemic status of *B. ovis* in Turkey [99].

Urgent measures should be taken to control ovine babesiosis. For the implementation of effective control strategies, diagnostic methods that are sensitive, specific, cost-effective, and suitable for use in the field should be developed. Furthermore, the presence of a vaccine would reduce the losses from outbreaks of the disease; thus, the development of vaccines is critical in this regard.

### 4.3. Equine Babesiosis

Equine babesiosis poses risks for horse breeding and racing industries in Turkey where international horse races are still held with growing interest. Most of the epidemiological surveys regarding this infection have been conducted to determine the prevalence of infection. The results of relevant researches indicate that *Theileria equi* is more prevalent compared to *Babesia caballi.* According to microscopical investigations, the prevalence of equine babesiosis varies from 0% to 58.18% [120,121,122,123,124]. Serologic studies indicated that the occurrence of *T. equi* varied from 12.8% to 64.5% and *B. caballi* from 0% to 34.6% [121,122,123,125,126,127,128,129]. Molecular prevalence was detected as 1.97% to 3% for *B. caballi* and 2.96% to 20.2% for *T. equi* [130,131,132,133]. However, Derinbay Ekici et al. [124] currently reported a high molecular prevalence for *B. caballi* (38.8%) and *T. equi* (50%) in tick-infested wild horses (*Equus ferus caballus*) from Central Anatolia in Turkey. Results from a comparative research revealed that equine piroplasm infections were more frequent in racehorses compared to stud-horses [127]. *Theileria equi* genotype A, D, E, and *B. caballi* genotype A have been detected in equids in Turkey [131,133].

Certain studies report that the occurrence of equine babesiosis differs according to the geographic region. Akkan et al. [121] detected that equine babesiosis was more prevalent in the eastern Turkey compared to other regions, with high positivity rates of 58.18% and 69.9% by microscopy and IFAT, respectively, which was attributed to the deficiency of regulations on animal movement across the border.

The information concerning the vectors of equine babesiosis is limited in Turkey. According to a few number of researches, *H. detritum, H. marginatum, R. bursa*, and *R. turanicus* were detected on horses with babesiosis [120,121,126]. Dik et al. [134] detected tick infestations caused by *Hae. parva*, *H. excavatum*, and *D. marginatus* in feral horses in Turkey. From an epidemiological point of view, most of these reports are not sufficient to elucidate the vector of the disease.

### 4.4. Canine Babesiosis

The epidemiology of canine *Babesia* species transmitted by *Dermacentor*, *Haemaphysalis*, and *Rhipicephalus* [81], is poorly studied in Turkey, but there has been an increase in the number of studies on this subject in recent years. Although tick-borne pathogens of pets are of economic importance in many developed countries [135], there are limited case reports indicating the occurrence of *Babesia* infections in dogs in Turkey [136,137,138]. Molecular studies investigating *Babesia* species showed the presence of *Babesia canis*, *Babesia gibsoni*, and *Babesia vogeli* in dogs in the country. The molecular prevalence of canine babesiosis was determined to be between 0% and 12% in these studies [139,140,141,142,143,144]. An unnamed novel *Babesia* spp. isolate has recently also been detected in dogs in Turkey [145].

## 5. Anaplasmosis

*Anaplasma* genus includes *Anaplasma bovis, A. centrale, A. marginale*, and *A. ovis* that are pathogens for ruminants, *A. platys* for canines, and *A. phagocytophilum* for domestic animals and humans. The main vectors of the *Anaplasma* species are ticks, particularly the genera *Ixodes, Amblyomma, Dermacentor*, and *Rhipicephalus* [146]. The direct transfer of *A. marginale*-infected erythrocytes from carrier animals to susceptible animals by biting arthropods or iatrogenically can result in the transmission of the disease. In Turkey, *A. marginale* infection is endemic with most animals being reservoirs.

### 5.1. Bovine Anaplasmosis

Bovine anaplasmosis is the most prevalent TBD of cattle initiated by *A. marginale* that causes a hemolytic disease. The disease can be fatal in susceptible cattle, and as such, partially responsible for the high mortality rate seen in affected herds. The clinical symptoms of the disease are anemia, fever, icterus, lethargy, abortion, weight loss, and often death in animals that are older than two years. Calves of immune mothers take temporary protection through the colostrum which prevents anaplasmosis. This protection lasts almost 3 months, and is mostly followed by an age resistance lasting until the animals are about 9 to 12 months. Animals recovered from acute infections become carriers of disease for life. Therefore, they significantly contribute to the spread of bovine anaplasmosis [147,148,149].

**Figure 4:**
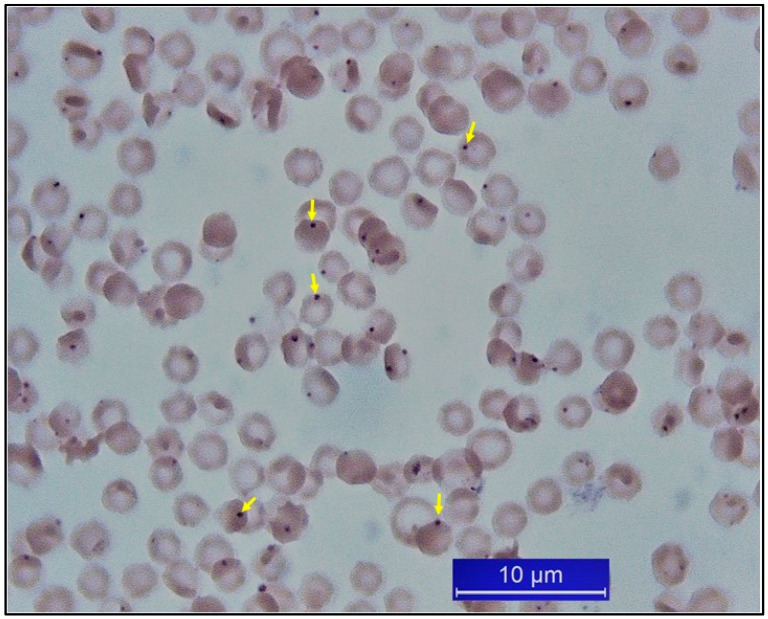
*Anaplasma marginale*-infected erythrocytes from a cattle (original).

Acute anaplasmosis is diagnosed using microscopy and identifying the infected erythrocytes on stained blood smears (Figure 4). During the acute infection, anemia occurs in a couple of days, and a sharp decline in the hematocrit indicates the severity of infection. During the persistent infection, the infected erythrocytes cannot always be detected in stained blood smears, and therefore, different serologic tests to detect specific antibodies are used for diagnosis. Competitive ELISA (cELISA) is generally preferred for this aim. It was reported that cELISA was positive in calves acutely infected with *A. marginale* before or along with the development of rickettsemia and that the antibodies were detectable in sera from persistently infected cattle vaccinated as long ago as six years previously [150].

Bovine anaplasmosis caused by *A. marginale*, *A. bovis, A. centrale*, and *A. phagocytophilum* has been reported in almost all regions of Turkey [23,151,152,153,154,155,156,157,158,159,160,161]. Most cattle represent the carrier state. In carrier animals, clinical infections may be developed as a result of immunosuppression usually related to pregnancy, transportation, and high milk yield. Deaths arising from acute infections have been recorded from both private [160] and governmental dairy cattle farms (unpublished data) in the country. Birdane et al. [155] reported fatal *A. marginale* infections in a dairy cattle herd from the central Aegean Region. In the herd, 34.11% and 55.35% of 645 animals were found positive with microscopy and cELISA, respectively, and fifteen cows died because of per-acute infection. Clinical cases were considerably associated with the age of the animals, as understood from the results. General microscopic and serologic surveillance in the herd showed cattle of all ages, except those aged 9 to 12 months and infected with *A. marginale*. Disease severity and the number of deaths increased with age. Seropositive calves aged younger than nine months did not exhibit any clinical symptoms of acute infection. Clinical bovine anaplasmosis cases caused by *A. phagocytophilum* were first reported by Aktas and Özübek [157] in 2015.

Imidocarb dipropionate and tetracyclines are commonly used drugs for the treatment of acute anaplasmosis. According to the reduction value in packed cell volume, the treatment regimen may be strengthened with blood transfusion and supportive therapy; however, death occurs in some per-acute cases despite all the treatment approaches [69].

### 5.2. Ovine Anaplasmosis

Anaplasmosis caused by *Anaplasma ovis* in goats and sheep is generally asymptomatic, but severe anemia may occasionally occur. The symptoms similar to those found in bovine anaplasmosis usually develop in the case of immunosuppression in small ruminants. *Anaplasma ovis* infection is transmitted by various ticks, particular *Dermacentor* and *Rhipicephalus* species, in Turkey mainly by *R. bursa* [44]. Clinical and subclinical infections in small ruminants were reported in a few studies by microscopic and serologic examination [8,99,151,162]. As a result of recently conducted molecular surveys, the prevalence of *A. ovis* was determined as 18% to 67.06% in Turkey [48,49,58,101,163,164,165,166].

Up to 2005, the occurrence of *A. phagocytophilum* in sheep was not reported in Turkey. In parallel to the development of TBDs in humans in recent years, *A. phagocytophilum*, which is thought to be zoonotic, was found in domestic animals and ticks [156,167,168,169,170]. The molecular prevalence of *A. phagocytophilum* in randomly selected sheep has varied between 0% and 18.90% in Turkey [48,49,101,164,165,168]. Benedicto et al. [58] detected the highest prevalence of *A. phagocytophilum* (66.7%) in tick-infested sheep and goats with clinical symptoms in Turkey. Further researches are required to obtain more epidemiological data concerning ovine anaplasmosis.

### 5.3. Canine Granulocytic Anaplasmosis and Cyclic Thrombocytopenia

*Anaplasma* species causing infections in dogs are *A. phagocytophilum* and *A. platys*. The diseases caused by these species are called canine granulocytic anaplasmosis and canine cyclic thrombocytopenia, respectively [171]. The main clinical symptoms of canine granulocytic anaplasmosis in dogs are fever, lethargy, lymphadenomegaly, anorexia, arthritis, splenomegaly, weight loss, and vomiting. *Anaplasma platys* frequently causes asymptomatic infections in dogs. Canine cyclic thrombocytopenia shows symptoms similar to canine granulocytic anaplasmosis, with the most prominent symptom being thrombocytopenia associated with anemia and leukopenia [172]. These diseases have a large distribution worldwide, including in Turkey. The serologic prevalence of *A. platys*/*A. phagocytophilum* was reported to range from 0% to 30.1% [173,174,175,176,177,178,179]. Several molecular studies have investigated the presence of these rickettsial organisms of dogs in Turkey [141,180,181,182]. The main tick vector of *A. phagocytophilum* is *I. ricinus*. However, apart from this tick species, *A. phagocytophilum* has been detected in *R. bursa* and *Hae. parva* in Turkey [170,181,183]. *Anaplasma platys*’ DNA has frequently been detected in *R. sanguineus.* For this reason, this tick species is considered the vector of *A. platys* in dogs [171]. *Anaplasma platys* was detected in *R. sanguineus* and *R. turanicus* in Turkey [181].

## 6. Ehrlichiosis

Ehrlichiosis is caused by obligately tick-transmitted rickettsial microorganisms belonging to the family Anaplasmatacea. *Ehrlichia canis*, *Ehrlichia chaffeensis*, *Ehrlichia ewingii*, *Ehrlichia muris, Ehrlichia mineirensis*, and *Ehrlichia ruminantium* are the main causative agents of the disease. However, the number of new species or genotypes of this genus is increasing as a result of developing molecular techniques [184,185]. *Ehrlichia canis, E. chaffeensis*, and *E. ewingii* are the most important species threatening human and animal health, especially in dogs. Severe canine infections are principally associated with *E. canis*, and the disease caused by this microorganism is called canine monocytic ehrlichiosis (CME) [186]. CME is mainly characterized by fever, anorexia, mucosal pallor, generalized lymphadenomegaly, depression, lethargy, and splenomegaly. Hypothermia may even occur in severely pancytopenic dogs [187].

The geographic distribution of *E. canis* shows parallelism with the presence of the primary vector tick, *R. sanguineus* [188]. This tick species transmits *E. canis* transstadially under field conditions [189] and intrastadially under experimental conditions [190] and distributed worldwide, particularly in tropical and subtropical zone countries, including Turkey [5,188].

Ehrlichiosis is one of the most common TBDs in Turkey. Clinical infections have commonly been encountered in dogs, but the disease is rarely found in different hosts [181,191]. Mylonakis et al. [187] stated that CME may be one of the main causes of life-threatening pancytopenia in dogs in *E. canis*-endemic countries as well as places such as Turkey and South East Asia. The presence of *R. sanguineus* throughout Turkey supports this view [5].

Studies conducted in Turkey have shown that *E. canis* is the only species detected in dogs [140,141,181,192,193,194,195,196]. CME is common in Turkey, and the prevalence of the disease has been detected by serologic and molecular investigations [140,197,198]. Serologic tests such as ELISA and IFAT used to detect anti-*E. canis* specific antibodies, show the seroprevalence of *E. canis* to be between 0% and 74% [174,176,177,179,181,198,199,200,201,202,203,204]; however, the molecular prevalence was determined to be between 0% and 39.3% [140,141,142,144,181,193,194,196]. The genetic diversity of *E. canis* was poorly studied in Turkey. Sequence and phylogenetic analysis of the Tandem Repeat Protein 36 (TRP36) revealed five Tandem Repeat sequences (TRs) indicating genetic diversity. By far, three different genotypes including Brazilian genotype, US genotype and a novel genotype similar to a previously detected genotype in humans from Costa-Rica were reported in Turkey [195].

Apart from *E. canis*, *Ehrlichia risticii* (*Neorickttsia risticii*), the causative agent of equine monocytic ehrlichiosis, specific antibodies have been detected in thoroughbred horses in Turkey [205]. *Ehrlichia* spp. Omatjenne strain was also detected in cattle [156]. Further, a clinical infection case caused by *E. canis* was reported in a cat in Turkey [191]. The first human ehrlichiosis case has been reported in Konya province of Turkey [206]. In addition, *E. canis* DNA was detected in *R. sanguineus*, *R. bursa*, *D. marginatus*, and *Hae. sulcata* ticks in Turkey [181,183]. Although the number of studies concerning CME has increased in recent years in Turkey, it is thought that more comprehensive and detailed studies are required. A human ehrlichiosis case recently reported from the central part of Turkey [206] reveals the importance of these zoonotic rickettsial microorganisms.

## 7. Hepatozoonosis

*Hepatozoon* species, apicomplexan protozoan parasites (Adeleorina: Hepatozoidae), cause infections characterized by moderate to severe symptoms including fever, lethargy, cachexia, anemia, hyperglobulinemia, weight loss, and anorexia in a broad spectrum of animals, particularly mammals and reptiles, but also birds and amphibians [207]. Hepatozoonosis is caused by more than 340 species, and the species of veterinary importance among these parasitize domestic and wild carnivores [208]. While *Hepatozoon canis* and *Hepatozoon americanum* are the main etiological agents of canine hepatozoonosis, feline hepatozoonosis is primarily associated with *Hepatozoon felis* [209,210]. Transmission occurs when a host animal orally ingests an infected tick harboring mature oocysts of *Hepatozoon* species [208]. *Amblyomma maculatum* and *R. sanguineus* sensu lato are the primary vectors of *H. americanum* and *H. canis*, respectively [211]. However, the oocysts of *H. canis* have also been described in different ixodid ticks infesting dogs, including *Haemaphysalis flava*, *Hae. longicornis*, *Amblyomma ovale, R. microplus*, and *R. turanicus* [212,213,214,215]. Transplacental transmission of *H. canis* has also been reported [216].

Hepatozoonosis was firstly reported in Turkey in 1933 [217]. Thereafter, the majority of conducted studies have been related to the clinical course, treatment, and epidemiology of canine hepatozoonosis [140,218,219,220]. Microscopic, serologic, and molecular studies show that the prevalence of canine hepatozoonosis varies between 0.5% and 54.3% in Turkey [140,141,142,143,145,193,221,222,223,224]. Considering that dogs from many different parts of the country were included in these studies, it is concluded that canine hepatozoonosis is endemic throughout Turkey.

Feline hepatozoonosis, which is mainly caused by *H. felis*, is not as prevalent as canine hepatozoonosis, and clinical infection was reported in a cat in only one study [225]. The agent causing infection in that study was molecularly confirmed as *Hepatozoon* spp. at the genus level; therefore, there is no study reporting feline hepatozoonosis caused by *H. felis* in Turkey.

*Hepatozoon canis*, *H. felis*, *H. ursi*, and *Hepatozoon* sp. MF genotypes have been detected in ticks and carnivores, including dogs, red foxes, and bears in Turkey [183,223,226,227,228,229] *Hepatozoon* sp. MF was detected in dogs in the central part of Turkey [143,223]. The presence of *H. canis* DNA was detected in red foxes by Orkun and Nalbantoglu [226]. Akyuz et al. [227] recently reported the infection of *H. ursi* in *Ursus arctos* (Turkish brown bears) from the eastern part of Turkey.

In studies investigating the pathogens carried by ticks, *H. canis* was detected in *R. sanguineus*, *R. turanicus*, *D. marginatus*, *Hae. parva*, *Hae. sulcata*, and *I. ricinus* in Turkey [183,222,226,228]. *Hepatozoon felis* was also detected in *R. sanguineus* [183]. Orkun and Emir [228] detected *H. felis* DNA in *Hae. parva* collected from Eurasian lynx. In addition, *H. ursi* was recently reported in *H. marginatum*, *R. turanicus*, and *I. ricinus* collected from brown bears in Turkey [228]. The detection of *Hepatozoon* species in different ticks does not mean that those tick species are vectors of this protozoa. To confirm this idea, further experimental studies are required [230].

## 8. Conclusions

In Turkey, the primary TBDs of veterinary relevance are theileriosis, babesiosis, and anaplasmosis in cattle, babesiosis in sheep and goats, equine piroplasmosis in horses, cytauxzoonosis in cats, and babesiosis and anaplasmosis in dogs. These infections have been well-known by veterinarians and farmers. The principal tick-borne pathogens are *A. marginale, B. bigemina* and *T. annulata* in cattles, *B. ovis* in sheep and goats, and *B. caballi* and *T. equi* in donkeys and horses in Turkey. These infectious agents may cause acute clinical infections and deaths in animals. The infections can emerge as co-infections, and frequently become complicated with the presence of other diseases, so struggling with infections get challenging. Effective drugs are available and used in the treatment of all these infections. Early diagnosis of the infections increases the rates of recovery, and in addition to the primary treatment regimen, supportive treatment is most essential. Acute infections can be diagnosed via microscopic examination, but carrier animals are still tough to determine by microscopy. IFA test has been commonly used to determine the carrier animals of *Anaplasma, Babesia*, *Ehrlichia*, and *Theileria* species for many years. A recombinant antigens termed rMSP5- and MSP5-specific monoclonal antibodies based cELISA is currently used to detect *Anaplasma* infections in animals. Veterinary practitioners usually treat animals according to their clinical diagnostic experiences under field conditions. PCR-based molecular techniques have also been utilized to detect TBDs. These diagnostic techniques are valuable investigation tools for certain studies. However, they are of little value for the practitioners, and laborious for the examination of large numbers of samples. It is necessary to develop diagnostic methods which are sensitive, specific, and practical for use even under field conditions.

Relevant studies have been mostly performed to determine the prevalence of infections in Turkey. Detailed studies are required in terms of risk factors and host–parasite–vector interactions. Additionally, the studies have mostly been undertaken in the provinces of Central and Eastern Anatolia Regions, and since Turkey is a wide country, it is still necessary to perform studies representing the entire country.
